# The Connected Steady State Model and the Interdependence of the CSF Proteome and CSF Flow Characteristics

**DOI:** 10.3389/fnins.2017.00241

**Published:** 2017-05-19

**Authors:** Fabian Metzger, Daniel Mischek, Frédéric Stoffers

**Affiliations:** ^1^Department of Neurology, Ulm University HospitalUlm, Germany; ^2^Institut Dr. Foerster GmbH & Co. KGReutlingen, Germany; ^3^Fakultät für Mathematik und Wirtschaftswissenschaften, Institute of Analysis, Universität UlmUlm, Germany

**Keywords:** cerebrospinal fluid, steady state, blood-brain barrier, blood-cerebrospinal fluid barrier, neuroimmunology, rostrocaudal gradient, protein diffusion, CSF proteome

## Abstract

Here we show that the hydrodynamic radii-dependent entry of blood proteins into cerebrospinal fluid (CSF) can best be modeled with a diffusional system of consecutive interdependent steady states between barrier-restricted molecular flux and bulk flow of CSF. The connected steady state model fits precisely to experimental results and provides the theoretical backbone to calculate the *in-vivo* hydrodynamic radii of blood-derived proteins as well as individual barrier characteristics. As the experimental reference set we used a previously published large-scale patient cohort of CSF to serum quotient ratios of immunoglobulins in relation to the respective albumin quotients. We related the inter-individual variances of these quotient relationships to the individual CSF flow time and barrier characteristics. We claim that this new concept allows the diagnosis of inflammatory processes with Reibergrams derived from population-based thresholds to be shifted to individualized judgment, thereby improving diagnostic sensitivity. We further use the source-dependent gradient patterns of proteins in CSF as intrinsic tracers for CSF flow characteristics. We assume that the rostrocaudal gradient of blood-derived proteins is a consequence of CSF bulk flow, whereas the slope of the gradient is a consequence of the unidirectional bulk flow and bidirectional pulsatile flow of CSF. Unlike blood-derived proteins, the influence of CSF flow characteristics on brain-derived proteins in CSF has been insufficiently discussed to date. By critically reviewing existing experimental data and by reassessing their conformity to CSF flow assumptions we conclude that the biomarker potential of brain-derived proteins in CSF can be improved by considering individual subproteomic dynamics of the CSF system.

## Introduction

The cerebrospinal fluid is a complex flow system likely involved in many brain-related tasks such as neuronal communication, waste clearance and immune surveillance of the brain (Abbott, 2004; Veening and Barendregt, 2010; Ransohoff and Engelhardt, 2012; Rodan et al., 2015). Its close proximity to the brain parenchyma and its delineation from the systemic circulation makes CSF a valuable source of information about brain-related changes. The CSF system is located in the ventricles (inner cavities of the brain), the central canal of the spinal cord and the area surrounding the brain and spinal parenchyma, the cranial and spinal subarachnoid space (SAS). The total fluid volume is around 150 ml at a production rate of ~0.3–0.5 ml/min. The CSF cushions the brain parenchyma and reduces the net weight of the human brain from ~1,500 to ~50 g (Cserr, 1971). In addition the fluid system acts as an important passive modulator of intracranial pressure (ICP) by equalizing cardiac cycle-driven blood volume changes inside the unyielding skull. During systole, the arterial blood inflow into the cranium exceeds the venous outflow, the compliant CSF fluid is pushed down the vertebrae and the lumbar sac dilates. The process is reversed during diastole (Wagshul et al., 2011). This leads to the bi-directional flow characteristics of CSF. The characteristics of pulsatile movement of CSF, depending on a variety of other physiological aspects such as breathing (Klose et al., 2000), or compliance, are more elaborately reviewed in Wagshul et al. (2011) and Linninger et al. (2016). Besides the generally accepted pulsatile flow characteristics; there is more controversial discussion on the bulk flow concept of CSF. The majority of scientists assume that the CSF is predominantly produced inside the ventricular system at the site of the choroid plexuses (CP) and absorbed into the lymph system and into the blood circulation in the subarachnoid space (SAS). In the cranial SAS CSF may partly flow from the SAS into the brain parenchyma via paravascular pathways and is then absorbed via the glymphatic system (Nedergaard, 2013). An excellent review focusing on the mechanisms of interstitial and CSF fluid movement is given in Hladky and Barrand (2014). For the purpose of this study it is sufficient to state that these assumptions presume a directed flow (bulk flow) from the ventricular space into the SAS. However, other researchers believe that CSF fluid is mainly generated and absorbed by the blood brain barrier (Brinker et al., 2014) and CSF movement is only a blood flow-dependent to-and-fro movement (Ore and Klarica, 2014). Although the majority of experimental results indicate a directed CSF flow, as reviewed in Abbott (2004), Damkier et al. (2013) and Spector et al. (2015), these experiments can be criticized based on their inherent invasiveness (Orešković and Klarica, 2010; Brinker et al., 2014).

The concentration of blood-derived proteins increases along the rostrocaudal axis whereas brain- or leptomeningeal-derived proteins remain relatively constant or possess a reverse gradient (Weisner and Bernhardt, 1978; Mollenhauer et al., 2012; Brandner et al., 2013, 2014; Aasebø et al., 2014; Sections The Rostrocaudal Gradient and Brain-Derived Proteins in CSF, **Table 3**). This is well explainable with the bulk-flow concept; blood-derived proteins enter the CSF system along the whole flow path of CSF, whereas brain-derived proteins predominantly enter the CSF system in the cranial area. Therefore, the concentration of blood-derived proteins rise with increasing distance from the ventricular system but the concentration of brain-derived proteins remains fairly constant along the spinal flow path (**Table 3**). With a pulsatile-only CSF flow concept, these subproteomic gradient characteristics are hard to explain. Further, although the pulsatile flow exceeds the bulk flow of CSF up to tenfold (Hladky and Barrand, 2014) and more (Gupta et al., 2009), the CSF flow remains laminar (Loth et al., 2001; Gupta et al., 2010) which is also indicated by the rostrocaudal gradient of blood-derived proteins, which would not exist in an CSF flow with predominantly turbulent flow characteristics. Therefore, evaluation of the source-related protein gradient values can be understood as intrinsic tracers for CSF flow characteristics.

Evaluating source-dependent protein characteristics can contribute to the understanding of the CSF system, but the consideration of the characteristics of the CSF system is of key importance for CSF proteomics.

For routine diagnostics CSF is withdrawn by lumbar puncture at the lumbar vertebrae. This gives rise to the questions as to how the brain-derived proteins are transported to the lumbar space (point of readout) and how their concentration is influenced by CSF dynamics (Section Brain-Derived Proteins in CSF).

Another key question is how blood-derived proteins are able to pass the barriers delineating blood from CSF (Section Discussion about the Diffusion Barriers). In the following sections we provide evidence that the passage of blood-borne proteins into CSF is at least predominantly based on diffusion (Section Blood-Derived Proteins in CSF), critically review a preceding diffusion-based model, the molecular flux model (Section The Molecular Flux Theory), and show that the diffusion-based protein exchange between blood and CSF can be precisely modeled with a system of steady states connected by bulk flow (Section The Connected Steady State Model).

## Blood-derived proteins in CSF

How precisely blood-borne proteins overcome the blood brain barrier (BBB) and/or the blood-cerebrospinal fluid barrier (BCSFB) remains to be shown on the physiological level, see (de Vries et al., 1997; Damkier et al., 2013) and Section Discussion about the Diffusion Barriers. However, it has been shown that the concentration quotient (*Q*) of blood-derived proteins in CSF (concentration CSF/concentration serum) is dependent on the hydrodynamic radius R_H_ of the protein and is lower for proteins with a higher hydrodynamic radius (Felgenhauer, 1974).

This is best studied with immunoglobulins in reference to albumin. Felgenhauer and Reiber showed that the relation of CSF to serum quotients of albumin to immunoglobulins (IgX = IgG/-A/-M) can be fitted with a hyperbolic function with the general formula:

(1) $$Q_{IgX}\;=\;\frac{a}{b}\sqrt{Q_{Alb}\;+\;b^{2}}-c$$

Equation (1) is derived from Reiber and Felgenhauer (1987) and claims that the *Q*-value ($\frac{c_{CSF}}{c_{blood}}$) of immunoglobulins can be expressed by the *Q*-value of albumin if the parameters a, b, and c are fitted correctly. Typically, the highly abundant protein albumin is chosen as the reference protein, but in principle all exclusively blood-derived proteins should be comparable by this method (Reiber and Felgenhauer, 1987). The hyperbolic function works well in the defined physiological range, but differently than stated by Reiber (1994a) this cannot have general validity since no combination of a, b, and c exists that can fulfill the Equation if one sets Q_*IgX*_ = Q_*Alb*_ (see also Appendix in Figure 5A).

**Figure 1:**
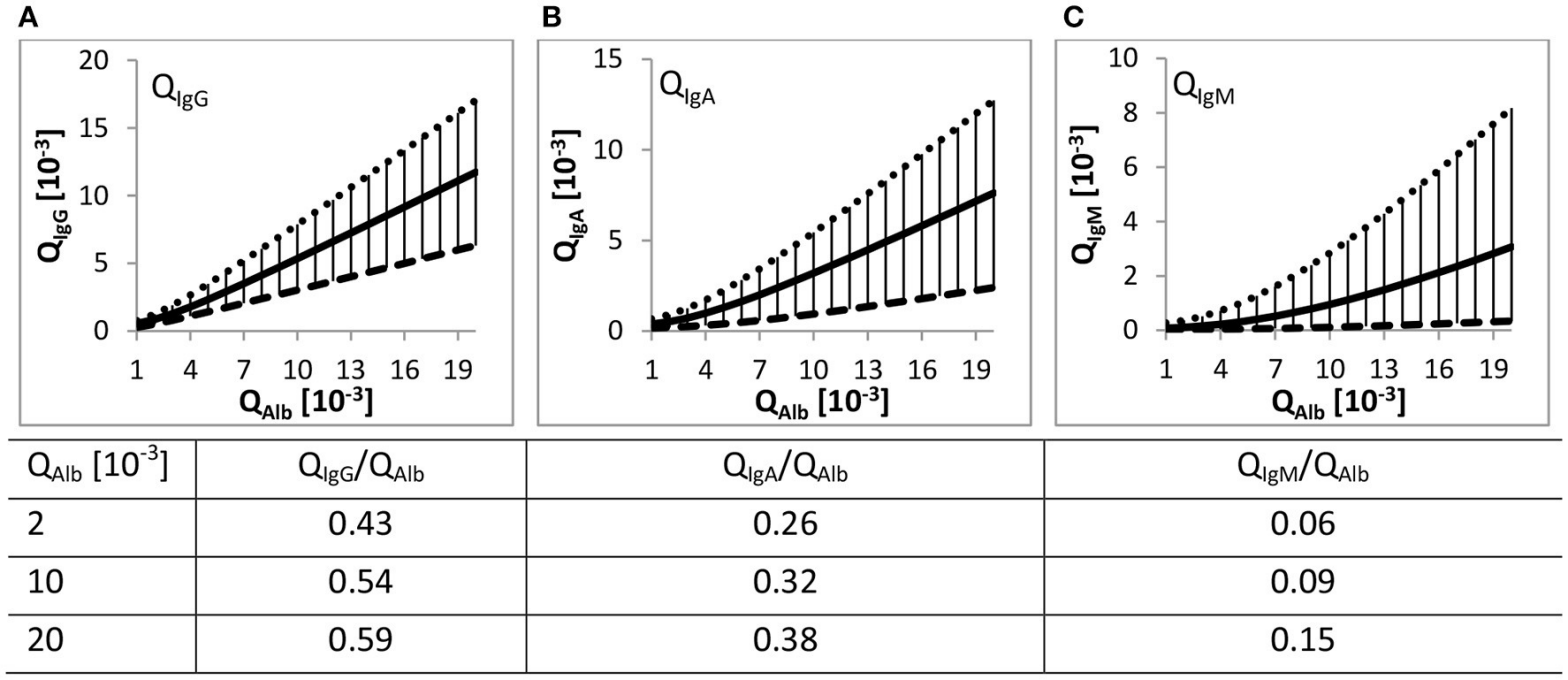
**Quotient gradient diagrams (Reibergrams)**. The gradient curves drawn with Equation (1) and values listed in Table 1, both derived from Reiber (1994a), represent the 99% interval of the Q_IgX_ to Q_Alb_ value relations from 4254 patients. **(A–C)** The continuous lines represent the mean gradient curve between Q_Alb_ and the corresponding Q_IgX_. The dotted and dashed lines are the upper and lower discrimination curves including 99% of all patient data. The lower curves of Q_IgG/A/M_ are ~ factor 2.5/4/20 distant to the respective upper hyperbolic function. The distance increases with increasing difference in R_H_ of albumin to the respective immunoglobulin. The vertical lines represent the range of the 99% intervals. Listed values are the $\frac{\text{QIgX}}{\text{QAlb}}$ ratios relative to the corresponding Q_Alb_ values.

The Reibergrams (Figure 1) with albumin as the reference protein are used to elucidate whether the occurrence of immunoglobulins in CSF is exclusively blood-derived or if there is additional contribution such as intrathecal immunoglobulin synthesis (Reiber and Felgenhauer, 1987). Values above the population-based determined upper hyperbolic function indicate inflammatory processes in the brain (Reiber and Peter, 2001).

In a follow-up study Reiber repeated the approach but with a considerably higher number of patients (4254 patients) (Reiber, 1994a). The high number of data points over a huge range of Q_Alb_ values allowed him to draw general conclusions. In short, with increasing Q_Alb_ the Q values of the considered immunoglobulins—IgG/-A/-M—also increase. Further, with increasing Q_Alb_ the absolute deviation of the mean hyperbolic function to the upper and lower discrimination line increases but the relative variation decreases or remains almost constant (see Table 1). Based on the high number of patient data this variation of Q_IgX_ to Q_Alb_ was termed population variation coefficient (Table 1).

**Table 1:** **Parameter values for the hyperbolic function and population variation coefficients for Q_**IgG/−A/−M**_ relative to Q_**Alb**_**.

	^*^Values for the hyperbolic functions				^**^Population Variation Coefficients $△{\text{Q}}_{\text{I}gX}/\bar{{\text{Q}}_{\text{I}gX}}$			
		**a/b**	b^2^^*^10^−6^	c^*^10^−3^	**Q_Alb_ [10^−3^]**	**IgG**	**IgA**	**IgM**
IgG	Upper limit	0.93	6	1.7	2.2	0.86	1.36	3
	Mean	0.65	8	1.4	3.5	0.89	1.41	3
	Lower limit	0.33	2	0.3	5	0.9	1.43	3.1
					8.2	0.92	1.42	2.9
IgA	Upper limit	0.77	23	3.1	10	0.91	1.4	2.9
	Mean	0.47	27	2.1	15	0.91	1.38	2.7
	Lower limit	0.17	74	1.3	20	0.91	1.34	2.6
					50	0.92	1.31	2.2
IgM	Upper limit	0.67	120	7.1	100	0.92	1.29	2
	Mean	0.33	306	5.7	140	0.92	1.29	2
	Lower limit	0.04	442	0.82				

The Table represents Table 1 and 2 in Reiber (1994a);

* *Values used with Equation (1) to draw Figure 1*.

** *Population variation coefficients are defined as upper minus lower hyperbolic curve divided by the mean curve at the given values of Q_Alb_. The population variation coefficient increases from Q_IgG_ to Q_IgA_ to Q_IgM_ according to the increased difference in hydrodynamic radius to albumin, indicating that the variation does not simply show the accuracy of the measurements with variation coefficients of <10% (Reiber, 1994b). With increasing Q_Alb_, the population variation coefficient remains constant for IgG but decreases in the case of IgA and IgM*.

At the same Q_Alb_ the specific Q_IgG/A/M_/Q_Alb_ ratio is dependent on the difference in R_*H*_ of the bigger immunoglobulins to the smaller albumin (see Table 2). IgG has the least difference in size to albumin and IgM the highest; therefore, at the same Q_Alb_, Q_IgG_/Q_Alb_ > Q_IgA_/Q_Alb_ > Q_IgM_/Q_Alb_ (values listed in Figure 1). Reiber concluded that this R_H_ dependency and the relative constancy of the population variation coefficients support the concept of a diffusion-driven process.

**Table 2:** **Relation of the diffusion coefficient to the hydrodynamic radii**.

**Quotient**	Q (R_H_)^−1^*^^	Q (ͽ)^**^	Q (B)^***^	Q (D_Mf_)^****^	**ΔMW [kDa]**
Albumin to IgG	1.5	1.62	1.08	1.23	~50
Albumin to IgA	1.85	2.32	1.25	1.42	~250
Albumin to IgM	3.6	3.6	1	1.82	~800–900

* *The experimentally measured hydrodynamic radii are: albumin = 3.51 nm, IgG = 5.29 nm, IgA = 6.50 nm (monomeric variant) and IgM = 12.65 nm derived from Armstrong et al. (2004)*.

** *Values were calculated with the values derived by fitting the steady state model to the mean hyperbolic functions, shown in Figure 3B (albumin = 2; IgG = 1.23; IgA = 0.86; IgM = 0.55)*.

*** *Q(B) is calculated according to Equation (16)*.

**** *The same approach for the molecular flux model as for the connected steady state model. Calculations are depicted in the Appendix (Equations A3/4 and Figure 5)*.

For instance, if the variation of Q_IgX_ to Q_Alb_ were based on individual variance of non-diffusional immunoglobulin transport from blood to CSF, then the population variation coefficient would decrease considerably with higher Q_Alb_ values. This is because the total variation would remain unchanged and not the variation coefficient (Reiber, 1994a). So far we follow his assumptions but in contrast to him we want to emphasize that the Q_IgX_/Q_Alb_ values increase with increasing Q_Alb_ (values in Figure 1). We further observed that the population variation coefficients of Q_IgA_ and Q_IgM_ decrease with increasing Q_Alb_ and that the extent of decrease seems to be dependent on the difference in R_H_ of IgX to albumin (Tables 1, 2).

## The molecular flux theory

Based on his observations, Reiber proposed a diffusion-based concept, the “molecular flux theory.” Although the basic ideas are intriguing and are fundamental for this work, the implementation contains several misconceptions leading to erroneous conclusions. He stated: “*The steady state between molecular flux into CSF and CSF flow rate determines the CSF concentration of a single protein”* and that the blood CSF system has the following boundary conditions “*[…] diffusion in a semi-infinite media with constant concentration at one surface […]”* (Reiber, 1994a). We concur with these assumptions, but surprisingly, he selected a nonlinear diffusion model without keeping the concentration constant at one boundary. The selected diffusion model with constant concentration c_0_ at the blood facing boundary yields:

(2) $$Q\;=\;\frac{c\left(x,t\right)}{c_{0}}\;=\;erfc\;\frac{x}{2\sqrt{\left(Dt\right)}}$$

In Equation (2) (Equation 2.45 in Crank, 1975), the constant concentration c_0_ equates to the concentration in blood, *c*(*x,t*) equates to the respective concentration in CSF, −∞ < x ≤ 0 equates to the semi-infinite media (blood), the boundary is at *x* = 0 and CSF has values of *x* > 0, *t* = time and *D* is the diffusion constant. Erfc, the error function complement is a standard mathematical function described in Equation 2.11 by Crank (1975). The conditions selected by Reiber (1994a) and Equation 2.14 in Crank (1975) yield:

(3) $$Q\;=\;\frac{c(x,t)}{c_{0}}\;=\;\frac{1}{2}\;erfc\;\frac{x}{2\sqrt{Dt}}$$

This has the consequence that the concentration at the boundary *c*(*x* = 0) = $\frac{1}{2}$ C_0_ for all *t* > 0. These boundary conditions do not meet the required conditions at the blood surface and this means that the concentration of a blood-derived molecule in CSF can never reach the blood concentration of this molecule. This in turn violates Fick's first law that postulates that a net diffusional flux exists as long as a concentration gradient exists.

(4) $$J\;=\;-D\frac{\partial c}{\partial x}$$

Equation (4) is Fick's first law of diffusion; it postulates that the “diffusion flux” J is proportional to the concentration gradient ($\frac{\partial c}{\partial x}$), where *D* is the diffusion coefficient, c the concentration of the diffusing substance and *x* the position. Another consequence of the selected model is that the molecular flux from blood into CSF increases with increasing c(CSF) as long as c(CSF) <0.5 c(blood). Reiber explained this increase in molecular flux with increased tissue concentration because of increased concentration in CSF (Reiber, 1994a). However, under the assumption of diffusional exchange between blood and CSF, the molecule concentration between both systems is a consequence of diffusion from blood to CSF and diffusion from CSF to blood.

## The connected steady state model

### Conception of the connected steady state model

**Figure 2:**
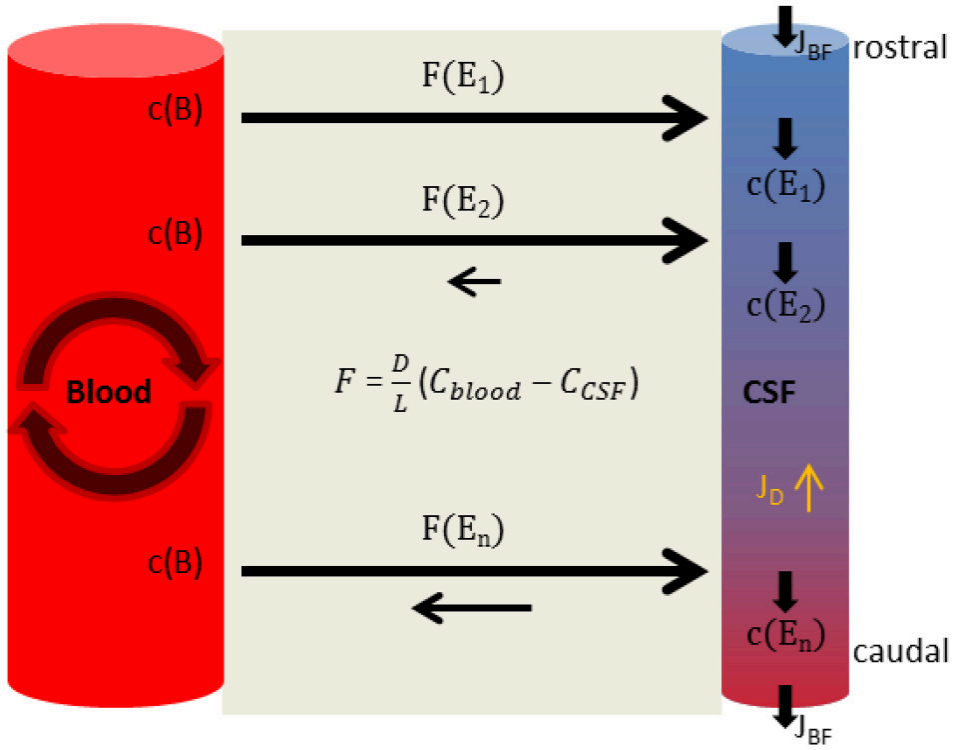
**The connected steady state model**. The concentration in blood, c(B), is constant. Diffusional loss is equalized by the circulative convection of blood. In consequence the diffusional flux from blood to CSF stays the same at each position. In the CSF system the diffusional uptake of blood-derived substances is in balance with the bulk flow of CSF J_BF_, so that the concentration at each position c(E_x_) remains constant but the concentration of blood-derived substances increases along the flow path of CSF–in consequence the diffusion of blood-derived proteins back to blood increases. At each position the molecular flux rate F(E_x_) equates to Equation (5) but is altered by the preceding steady states. The diffusional flux (J_D_) of blood-derived substances inside the CSF system is inverse to the bulk flow.

The diffusional loss of blood-derived proteins into the CSF system is in steady state with the bulk outflow of the CSF into blood. Furthermore, the blood volume of ~5000 ml is much larger than the CSF volume of ~150 ml. In addition, the fast circulative convection of blood superimposes the low barrier-restricted diffusional protein exchange with CSF. In view of this consideration it is feasible to state that the protein concentration in the blood system is constant. In contrast, the rostrocaudal CSF bulk flow equates to a river-like start-end point system with a rostrocaudal concentration gradient of blood-derived proteins. Both flow systems, CSF and blood, exist continuously; the molecular flux and the CSF bulk flow balance each other out. In conclusion, and as already stated by Reiber (1994a), each point along the neuraxis corresponds to a steady state between concentration increase due to effective inward diffusion from the blood into the CSF and concentration decrease due to bulk flow of CSF (Figure 2). Since no time dependence for the diffusional exchange exists, nonlinear model assumptions as stated in the “molecular flux theory” (Section The Molecular Flux Theory) must be wrong. The high perfusion of the spinal cord (Yoshizawa, 2002) allows the CSF to be seen as a cylinder surrounded by another cylinder—blood; thus we assume radial symmetry. Under that perspective, the diffusional exchange between the systems can be treated as one-dimensional diffusional exchange between two parallel planes under steady state conditions. According to Crank Chapter 4, Equation (5.4) (Crank, 1975) we obtain:

(5) $$F\;=\;-D\text{ dC}/\text{dx }=\;D(C_{1}-C_{2})/L$$

where *L* is the length distance between the concentrations *C*_1_ and *C*_2_ (blood and CSF) and *D* is the diffusion coefficient. The concentration gradient dC/dx along *L* is constant, whereas *C* decreases linearly over *L*.

According to Fick's first law (Equation 4), the molecular flux inside the CSF system (J_*D*_) is proportional to the concentration gradient and therefore inverse to the bulk flow of CSF. In a continuous system like the CSF system, the molecular flux inside the systems is also under steady state conditions. The J_*D*_ reduces the bulk flow (J_BF_) induced rostrocaudal gradient of a molecule proportionally to the diffusion constant of that molecule.

For a rough estimate of the inverse influence of J_D_ on the rostrocaudal gradient, the following physiologically orientated values are considered: The length of the flow path (L_FP_) from ventricle vault to lumbar sac is set to L_FP_ = 50 cm. For the volume of CSF, we ignore the cranial SAS volume and for the remaining volume of the CSF system we set V_CSF_ = 100 ml (Akdogan et al., 2010; Edsbagge et al., 2011). For reasons of simplification we interpret the CSF system as a tube with constant diameter and further assume that the CSF bulk flow velocity (*V*_*BF*_) equals the CSF production rate of 0.3 ml/min along the whole flow path so that $V_{BF}=\frac{0.3\frac{ml}{min}*50cm}{100ml}\;=\;0.015\frac{cm}{min}\;=\;0.0025\frac{cm}{sec}$. For the diffusion coefficient of the proteins we assume $D\;=\;10^{-6}\frac{cm^{2}}{sec}$ (Torres et al., 2012). This yields according to the Péclet number $Pe\;=\;\frac{V_{BF}*L_{FP}}{D}\;=\;1.25*10^{5}$ >> 1.

In conclusion, the molecular bulk flow is far greater than the molecular flux inside the CSF system. This allows diffusional fluxes inside the CSF system to be ignored.

Here we discuss the case of diffusion through an R_H_-selective barrier (Felgenhauer, 1974) with otherwise unclear and partly individual characteristics. For the purpose of this work it is sufficient to interpret the barrier structures, separating CSF from blood, as variable factor (B) which reduces the random thermal motion-based exchange between the systems. This factor (0 < B <1) is dependent on the individual barrier and specific molecule properties. Therefore, the resulting diffusional exchange is dependent on the diffusion coefficient of the specific molecule and the barrier properties; this yields the coefficient for diffusional exchange through a barrier:

(6) ͽ:​= D ∗ B

### Formulation of the connected steady state model

Mathematically the concentration of diffusing substances at the most upstream position in CSF is zero, and the resulting steady state molecular flux at this position is:

(7) $$F_{E1}\;=ͽL^{-1}C_{blood}$$

where *E*1 is the virtually spatial coordinate for the most upstream position in the CSF system. Every diffusional increase in concentration in CSF at this position is equalized due to the bulk flow of CSF. However, the concentration infinitesimally downstream of the starting point is:

(8) $$C(E1)\;=\;\frac{ͽL^{-1}\;C_{blood}}{v}$$

where *v* is the bulk flow velocity of CSF. The resulting molecular flux at this position is then:

(9) $$F_{E2}\;=ͽL^{-1}\;(C_{blood}\;-\;k^{\prime }\;ͽ\;L^{-1}C_{blood})$$

**Figure 3:**
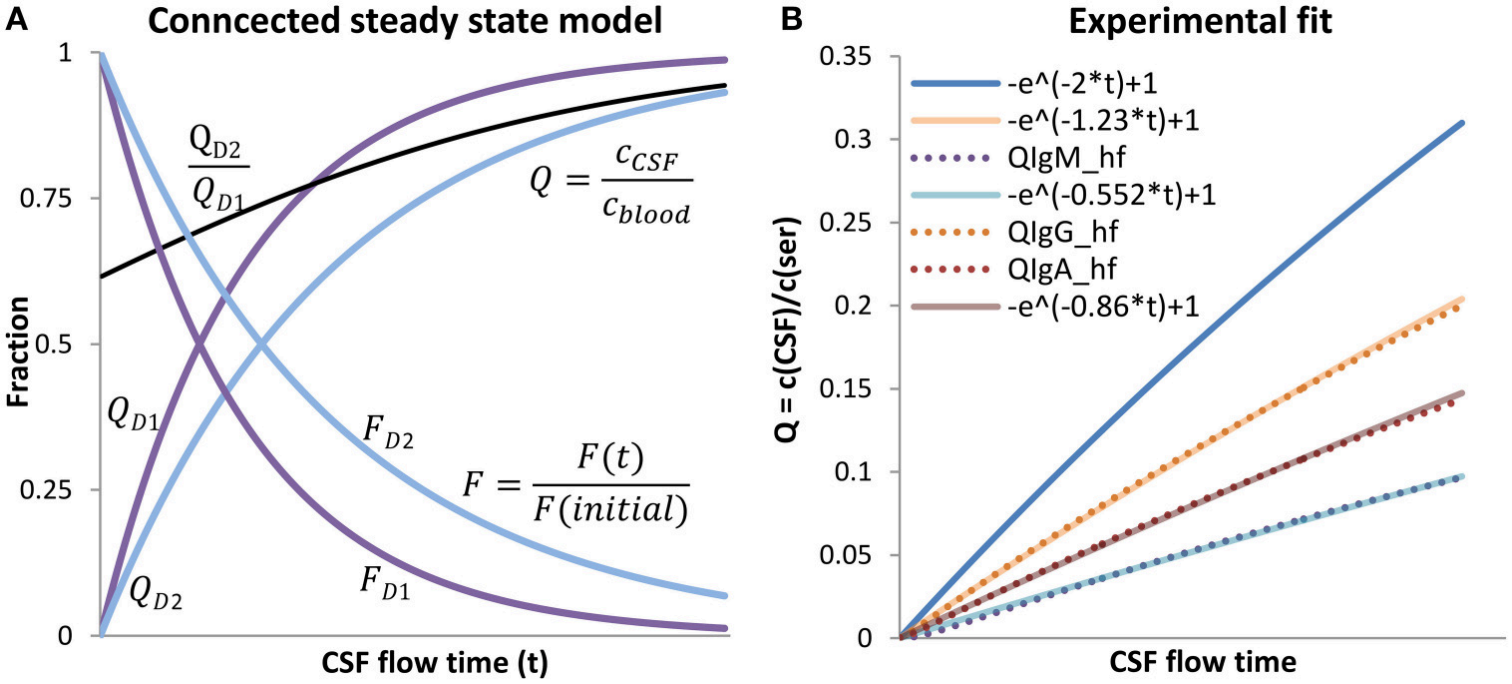
**The connected steady state model. (A)** The steady state model is shown for two different substances D1 and D2. For the faster diffusing molecule, D1, the resulting $\bar{ͽ}$ was set to 2 and for D2 to 1.23, k/L was set to 1. T was left variable and interpreted as CSF flow time (x-axis). Q_D1/2_ are drawn with Equation (10), F_D1/2_ with Equation (11). The black line is the gradient $\frac{Q_{D2}}{Q_{D1}}$ with the characteristic that $\frac{{\text{Q}}_{D2}}{Q_{D1}}\to 1$ when *t* → ∞. The concentration in CSF, relative to the blood concentration $\;\frac{c_{CSF}}{c_{blood}}$ of the slower diffusing substance D2, increases more slowly in CSF. With longer CSF flow times, the “molecular flux,” relative to the maximal rate $\frac{F\left(t\right)}{F\left(initial\right)}$, decreases faster for the faster diffusing substance D1 than compared to the slower diffusing substance D2, this is because of the faster increasing back-diffusion rate from CSF to blood of the faster diffusing substance D1. As a consequence, the gradient $\frac{Q_{D2}}{Q_{D1}}$ (black line) between the faster (D1) and the slower diffusing substance (D2) increases constantly toward 1; the different impression by observing Q_D1_ to Q_D2_ is caused by the scaling. The proportion of D1 to D2 equates to the fitted relationships of Q_Alb_ to Q_IgG_ in **(B)**. **(B)** Shows the experimental fit for the three immunoglobulins. Q_Alb_ is defined as Q_Alb_. The experimental data representing curves Q_IgG/−A/M_-hf were calculated using Equation (12) with the appropriate parameters for IgG/-A/-M (Table 1) The connected steady state equations were then manually fitted to the experimental data with Equation (13), by adjusting the ratio $\frac{\bar{{ͽ}_{IgX}}}{\bar{{ͽ}_{ALB}}}$.

Where $k\prime \;=\;\frac{1}{v}$. The mathematical solution of the diffusion problem, depicted in Appendix A1, results in the Equation for the change of concentration in CSF as a function of time:

(10) $$Q\;=\;\frac{C_{CSF}(t)}{C_{blood}}\;=\;-e^{-ͽ\frac{k}{L}t}\;+1$$

where *k* is the constant of proportionality with the unit m^−1^ and *t* the *CSF* flow time. *L* and ͽ have the same meaning as in Equations (5, 6). And analogous for the molecular flux:

(11) $$F\;=\;\frac{F_{CSF}(t)}{F\left(initial\right)}\;=\;e^{-ͽ\frac{k}{L}t}$$

where *F(initial)* is the maximum diffusion rate from blood to CSF, without back-diffusion. Since ͽ is a composite of a general value—D—and an individual value—B—, in the following ͽ is replaced by $\bar{ͽ}$ containing the mean value for the barrier B. The course of the two functions (Equations 10, 11) in dependence to CSF flow-time is shown in Figure 3A.

### Validation of the connected-steady state model

To test if Equation (10) is able to explain the experimental data shown in Figure 1, Equation (1) is rewritten with $e^{\bar{{-ͽ}_{Alb}}\frac{\text{k}}{L}t}$ replacing Q_*Alb*_:

(12) $$\bar{Q_{IgX}}\;=\;\frac{a}{b}\sqrt{e^{-\bar{{ͽ}_{Alb}}\frac{\text{k}}{L}t}\;+\;b^{2}}-c$$

If the steady state system is able to explain the experimental data, then the curve of Equation (12) must be similar in the physiological range to:

(13) $$\bar{Q_{IgX}}\;=\;e^{-\bar{{ͽ}_{Alb}}\frac{\text{k}}{L}t\;*\;\frac{\bar{{ͽ}_{IgX}}}{\bar{{ͽ}_{ALB}}}}\;+1$$

Equation (12) represents the experimental data and Equation (13) the theoretical assumption that the difference of Q_IgX_ and Q_Alb_ is based on diffusion and molecule-dependent barrier properties. A similar approach was used for the molecular flux concept by Reiber (Table 5 in Reiber, 1994a) but in that study the hyperbolic function was fitted to Equation (3) using arbitrary values. Here we fit the theoretical Equation (13) to the empirical Equation (12) by setting the parameter values for a, b, and c according to the experimentally derived values for IgG/A/M derived from Reiber (1994a), listed in Table 2.

For $\bar{{ͽ}_{\text{Alb}}}$ we set the arbitrary value at 2 and for $\frac{\text{k}}{L}$ at 1. It should be noted that the values for $\bar{{ͽ}_{Alb}}$ and $\frac{\text{k}}{L}$ are arbitrary values, which is permitted since the only demand for the fit to the experimental data representing hyperbolic function is that the values for Q_Alb_, calculated by Equation (10), have to represent physiological values. Figure 3B shows that in the physiological range the curve derived by Equation (13) fits precisely to the curve derived by Equation (12). A continuative discussion about the fitting procedure is given in the Appendix A2 (Figure 5).

### Interpretation of the connected steady state model

The connected steady state system as displayed in Figure 3A explains the increasing *Q*-values with an increase of the mean CSF flow time (*t*). With longer CSF flow time (*t*) the molecular flux (*F*) from blood to CSF decreases due to an increase in diffusion back from CSF to blood. Since the decay of the net molecular flux (*F*) is faster for the faster diffusing protein albumin, the ratio $\frac{Q_{IgX}}{Q_{Alb}}$ increases with increasing CSF flow time. However, the variation of $\frac{Q_{IgX}}{Q_{Alb}}$ at specific Q_ALB_ values, shown in Figure 1, cannot be explained by CSF flow time alone. In the case of free diffusion, *B* = 1, the quotient $\frac{{ͽ}_{IgX}}{{ͽ}_{Alb}}\;=\;\frac{D_{IgX}}{D_{Alb}}*\frac{B_{IgX}}{B_{Alb}}$ reduces to $\frac{D_{IgX}}{D_{Alb}}$. This means the $\frac{Q_{IgX}}{Q_{Alb}}$ ratio is only dependent on D, leaving no space for individual variations. In the case of diffusion through a barrier as in the considered blood-CSF system the value for B is dependent on the individual barrier properties. This means the relation $\frac{B_{IgX}}{B_{Alb}}$ is variable between individuals, and therefore the variation depends on *t* and $\frac{B_{IgX}}{B_{Alb}}$. This explains the physiologically high $\frac{{ͽ}_{IgX}}{{ͽ}_{Alb}}$ variation at specific Q_Alb_ values (Figure 1).

In Newtonian fluids the diffusion coefficient is related to the hydrodynamic radius via the Stokes-Einstein relation:

(14) $$D\;=\;\frac{{k_{B}}^{*}T}{6\pi^{*}\eta^{*}R_{H}}$$

where *D* is the diffusion coefficient, *k*_*B*_ the Boltzmann constant, η the viscosity of the medium, *T* the temperature and *R*_*H*_ the hydrodynamic radius of the molecule. Here we discuss the case of diffusion through a barrier. We assume that for the barrier restricted diffusional exchange the physical principles stay the same. With Equation (6) we obtain:

(15) $$ͽ\;=\frac{k_{B}*T}{6\pi *\eta *R_{H}}*B$$

The molecule-specific influence of the barrier on the molecular flux is contained in B. This allows a comparison of the quotients of the hydrodynamic radii of albumin and immunoglobulins to the quotient value $\frac{\bar{{ͽ}_{IgX}}}{\bar{{ͽ}_{Alb}}}$ according to:

(16) $$\frac{\bar{{ͽ}_{Alb}}}{\bar{{ͽ}_{IgX}}}\;=\;\frac{R_{H(IgX)}}{R_{H\left(Alb\right)}}*\frac{\bar{B_{Alb}}}{\bar{B_{IgX}}}$$

All other possible factors such as η or *T* have the same influence on all molecules in the same individual. For albumin as well as for the immunoglobulins G/A/M experimentally measured R_*H*_ values are available. Table 2 shows the comparison of Q(R_H_)^−1^ values to the related Q(ͽ) values derived from Figure 3B.

For IgG to albumin, the quotients between ͽ and ${\text{R}}_{\text{H}}^{-1}$ are fairly similar but the *Q*(ͽ) is slightly increased. This trend is continued when comparing IgA to albumin but not IgM to albumin; the calculated relations are identical. A possibility for the difference in the case of IgA might be that we used the experimentally measured values for the monomeric variant of IgA, but IgA also exists as a dimer. However, in human serum IgA is dominantly monomeric (Woof and Kerr, 2004). Due to the R_H_-dependent barrier strength (B), *Q*(ͽ) deviates the more from Q(R_H_)^−1^ the more the difference in R_H_ of two compared molecules. In the case of IgM relative to albumin, the effect is superimposed by the fact that IgM also exists in variants with a lower R_H_ than used in Table 2 (Felgenhauer, 1974).

The coincidence of Q(R_H_)^−1^ and Q(ͽ) (Table 2) supports first the validity of the connected steady state model for the diffusional exchange between CSF and blood and second the assumption that the hydrodynamic radius is the dominant molecule-specific factor in explaining different Q values ($\frac{c_{CSF}}{c_{blood}}$) for blood proteins (predominantly globular and hydrophilic) in the same individual (Felgenhauer, 1974). This might not be transferable to other molecule classes, e.g., predominantly lipophilic substances.

In contrast, the molecular flux theory predicts Q(D_Mf_) values lower than Q(R_H_)^−1^ values (Table 2). In the case of free diffusion (Equation 14), Q(R_H_)^−1^ = Q(D), and therefore Q(D) < Q(R_H_)^−1^ is not possible. In the case of barrier-restricted diffusion; Q(ͽ) < Q(R_H_)^−1^ is possible since other molecule-specific characteristics included in B, e.g., polarity, may play a superior role to R_H_, however, as mentioned, this does not seem to be the case for blood proteins and in addition the molecular flux theory describes a scenario for unrestricted diffusion and does not contain any correction factor representing barrier characteristics (like B in the connected steady state model); therefore Q(D) < Q(R_H_)^−1^ is not possible and shows again that the diffusion concept used for the molecular flux theory is not applicable for the diffusional exchange between CSF and blood under steady state conditions.

### Applications of the connected steady state model

Under the assumption that molecule-specific characteristics other than R_H_ can be ignored a deduction from the model is that in the case of proteins with the same R_H_ no variance between a Q_x_/Q_y_ value in different individuals exists and that the Q_x_/Q_y_ ratio is always 1. This allows the calculation of the *in vivo* R_H_ of blood-derived proteins by the evaluation of their Q values relative to the Q values of a set of reference proteins with known R_H_ according to Equation (16). Proteins not adjustable to a reference protein, with remaining high variances, might possess several R_H_ values such as IgM. Care must be taken with proteins in CSF not only derived from blood but at the other site; this fraction can be calculated if their R_H_ is known.

A direct clinical application is the diagnosis of inflammatory processes in the CNS. Also because of the high variation of the Reibergrams (Figure 1), oligoclonal bands are used as additional diagnostic criteria (Davenport and Keren, 1988). We predict that the diagnostic sensitivity can be improved by selecting reference proteins with the same or a similar hydrodynamic radius as the immunoglobulin of interest. This makes the diagnostic approach insensitive to individual barrier properties and allows a shift from population-based thresholds to absolute values.

Another deduction from the model is that by comparing a set of reference molecules with known R_H_ the R_H_-dependent barrier specificity can be calculated. As an example, if the Q(B) of two molecules with R_H_ of 3 and 4 nm is greater than the Q(B) of two proteins with R_H_ of 4 and 5 nm, then the conclusion is that the barrier is more discriminative between 3 to 4 nm than between 4 and 5 nm. This allows conclusions on the physiological properties of the barrier to be made.

### Physiological considerations

The “molecular flux theory” (Reiber, 1994a; Section The Molecular Flux Theory) claims that pathologically high Q_Alb_ values can be explained by an altered CSF flow rate without the need of assuming a change in barrier permeability. The physiologically normal Q_Alb_ in lumbar CSF is around 2–8 [10^−3^]. This value increases in an age-dependent manner (Reiber et al., 2001) whereas CSF turnover decreases from 3–4 times per day in young adults to less than twice in the elderly (Smith et al., 2015).

According to the steady state Equation (10), at this low Q_Alb_ the change of $Q\left(\frac{c_{CSF}}{c_{blood}}\right)$ is almost linear to the change of CSF flow time (Figure 3A), so that a doubling of CSF flow time means a doubling of the Q_Alb_ values, e.g., from 3.5 [10^−3^] to 7 [10^−3^]. This fits very well to the change of Q_Alb_ and CSF turnover from young adulthood to the elderly. The same calculation done with the molecular flux theory (Equation 3)—a turnover reduced by half and therefore assuming a doubled CSF flow time—yields an increase of Q_Alb_ e.g., from 2 [10^−3^] to 20 [10^−3^] ($\frac{1}{2}\;erfc\;\left(2.035\right)=0.002$ and $\frac{1}{2}\;erfc\;\left(1.45\right)=0.02$ 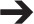 ${\left(\frac{2.035}{1.45}\right)}^{2}=1.96$ 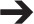 change of CSF flow time). Such a high sensitivity of Q_Alb_ to CSF flow time is rather unlikely.

A Q_Alb_ > 8 [10^−3^] is termed as barrier dysfunction. The increase from 2 [10^−3^] to 20 [10^−3^] calculated with the connected steady state system would mean a roughly tenfold increase in CSF flow time. With the current physiological understanding of the CSF flow system, a tenfold increase of CSF flow time seems to be a rather large change.

In conclusion, the normal age-dependent increase of Q_Alb_ is mainly dependent on reduced CSF turnover and not based on changed barrier permeability. But pathophysiologically high Q_Alb_ values indicate a pathophysiological change of the barrier permeability.

Reiber interpreted the population variation coefficients (see Section Blood-Derived Proteins in CSF) as constant and therefore he concluded that the barrier specificity remains unchanged which indicates unchanged barrier permeability (Reiber, 1994a). We interpret the population variation coefficients (Table 1) as values slightly decreasing with increasing Q_Alb_ and therefore assume that the barrier specificity slightly decreases with increasing Q_Alb_. Regarding barrier permeability and R_H_-dependent discrimination Felgenhauer speculated: “*Holes are formed by random thermal motion of the membrane structure elements and the permeability properties are characterized by formation frequencies and size probability distributions of these holes.”* (Felgenhauer, 1974).

**Figure 4:**
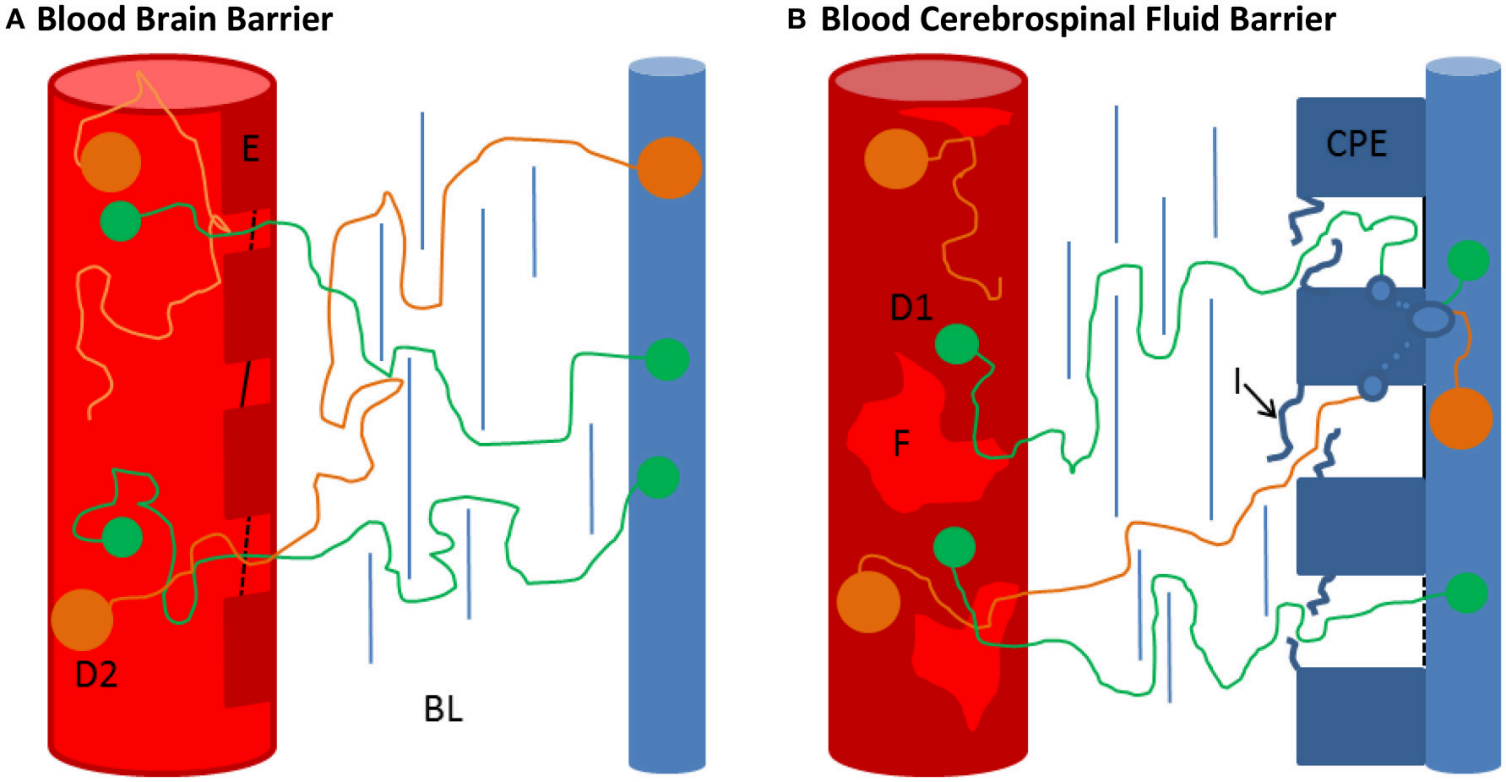
**Comparison of the BBB and the BCSFB. (A,B)** Schematic representation of the blood brain barrier (BBB) and the blood-CSF-barrier (BCSFB) (not to scale). **(A)** The BBB is composed of a basal lamina, embedding endothelial cells (E), pericytes and perivascular macrophages, and astrocytic end feet (de Vries et al., 1997). The continuous brain capillaries (red cylinders) are fully covered by cerebral endothelial cells (E). These cerebral capillary endothelial cells are closely connected by tight junctions (black lines), localized close to the lumen of the capillaries (de Vries et al., 1997). Further, fluid phase uptake of the endothelial cells seems to be very limited as pinocytotic activity is hardly observed (de Vries et al., 1997). Physiologically there is no obvious explanation of how the proteins manage to pass the BBB. However, taking into account that under normal physiological conditions almost no diffusion occurs (Q_Alb_ ~ 0.004) although the length of the barrier is in the lower μm range, Felgenhauers ‘hole’ concept becomes plausible (see text). For the smaller molecule D1 smaller holes are sufficient to overcome the barrier than for the bigger molecule D2. Also the passage through the basal lamina (BL) and glia limitans might be less hampered for the smaller molecule. Individual variations in these physiological areas might explain different Q_IgX_/Q_Alb_ ratios at a specific Q_Alb_value. **(B)** In contrast to the BBB the BCSFB possesses fenestrated capillaries (F), the tight junctions are at the CSF-facing side (blue cylinder) and vesicles are commonly observed in the choroid plexus epithelial cells (CPE) (Damkier et al., 2013; Strazielle and Ghersi-Egea, 2013). The uptake of blood-derived substances in the CPE seems to take place at the intercellular spaces of the CPE (van Deurs et al., 1978; Lu et al., 1993). At first glance, the R_H_ dependency of protein passage makes a transcellular route unlikely. However, the fenestrated diaphragm-coated capillaries of the choroid plexuses, the interdigitations (I) of the lateral basal lamina and other junctional complexes might be feasible barriers before the capillary-derived fluid is taken up by the CPE. This might explain the ostensible discrepancy between the size dependency of blood-derived protein passage and transcellular passage.

Following this speculation helps to imagine how individual differences in the barrier properties lead to differences in R_H_-dependent discrimination. A weaker barrier might have a weaker discrimination between molecules with different hydrodynamic radii (see Figure 4). Thus, an increased Q_Alb_ means an increase in flow time and/or a decrease in barrier permeability and specificity.

However, relative to the high differences in Q_Alb_ (Figure 1) the barrier specificity indicated by the population variation coefficients (Table 1) remains relatively stable, which indicates that barrier permeability and specificity are partly uncoupled.

## Discussion about the diffusion barriers

The CSF system is separated from the blood by the cerebrospinal fluid barrier (BCSFB) at the site of the CP, the blood brain barrier (BBB) which makes up 99% of all brain capillaries (de Vries et al., 1997) and the blood-meningeal barrier (BMB, alternatively referred to as outer BCSFB) which separates the SAS from the perfused dura, the outermost meningeal layer. The brain capillaries and the blood vessels in the SAS are enclosed by endothelial cells closely tied by tight junctions with pore sizes of only up to 1 nm, therefore attributing main barrier functions closely to the vasculature (Nabeshima et al., 1975; Sarin, 2010). In contrast, the capillaries of the CP and partly in the dura are fenestrated, providing pore sizes of up to 12 nm; which is also the approximate diameter of the 800 kDa protein ferritin, and therefore allowing passage of proteins (Strazielle and Ghersi-Egea, 2013). The main barrier function is attributed to the CSF-facing side, by the arachnoid barrier cell layer in the BMB and the epithelial cells of the CP (CPE) in the BCSFB, both barriers are tightly sealed by tight junctions, revealing no obvious space for diffusional fluxes for molecules in the size range of proteins (Vandenabeele et al., 1996; Barshes et al., 2005; Damkier et al., 2013). In contrast to the other barrier structures, a transcellular passage is commonly observed at the CPE and is therefore seemingly the main exchange route between CP and CSF (Becker et al., 1967; Damkier et al., 2013). This questions the observed size dependency of blood-borne protein entry at this barrier site but possibly size differentiation occurs via a staggered mechanism of preceding barrier structures (see Figure 4). At the other barriers transcellular passage is commonly not observed which questions how proteins are able to diffuse from blood to CSF at all at these sites. More elaborate descriptions of the morphology and function of the barriers are given for instance in de Vries et al. (1997), Barshes et al. (2005), Redzic (2011), Damkier et al. (2013) and Strazielle and Ghersi-Egea (2013). For this work, it is sufficient to conclude that the physiological mechanism allowing diffusional exchange remains to be clarified.

Regarding the influence of the barriers the following physiological aspects have to be considered. The total volume of CSF ~150 ml divides into ventricle CSF ~25 ml and SAS ~125 ml (Sakka et al., 2011). At a CSF synthesis rate of 0.4 ml/min, this results in a mean residence time in the ventricle CSF of ~1 h and ~5 h in the SAS. Since beyond the ventricles, the CSF flow divides into the cranial and the spinal SAS flow and the volume flow in the spinal SAS is further reduced continuously by CSF outflow along spinal nerve roots (Pollay, 2010) reducing the bulk flow velocity to almost zero at the lumbar area (Greitz and Hannerz, 1996), the flow time in the SAS might be considerably longer than indicated by this calculation. Even so, the concentration of blood-derived proteins in ventricle CSF is almost half the concentration of lumbar CSF (Weisner and Bernhardt, 1978; Reiber, 2001; Table 3). A possible explanation might be that the barriers delineating CSF from blood have different properties (Figure 4). Intriguingly, the population variation coefficients (Table 1) first increase up to Q_Alb_ ~ 5 [10^−3^] and then decrease continuously. Potentially the impact of the presumably lower restrictive barrier of the BCSFB inside the ventricles is higher at lower Q_Alb_-values because of shorter spinal flow time of CSF. However, at lower Q_Alb_-values, the Q_IgG_ to Q_Alb_ values are lowest (Figure 1). This supports the general assumption that the barrier is more restrictive at low Q_Alb_ values. There might be a break-even point between the influence of the BCSFB and the other barriers. However, since we used the fitted hyperbolic functions (Table 1, Figure 1) as the representation of the experimental data, we are not aware how well the fit worked in the lower physiological Q_Alb_ range (see Appendix in Figures 5A,B).

**Table 3:** **Source-dependent protein concentration change along the flow path of CSF**.

**Comparison**	**Lateral ventricle CSF/lumbar CSF**				**Spinal rostrocaudal gradient: R/C**			
**Study**	**Brandner et al**.^*^		Reiber^**^		**Mollenhauer et al**.^***^		**Aasebø et al**.^****^	
Brain proteins	Tau	6.83^*I^	S100B	3.5	NSE	1.13	P05060	0.96
		0.97^*II^	NSE	2			Q06481	1.01
Albumin	0.56^*I^; 0.52^*II^		0.4		0.56		0.63	

*Lateral ventricle CSF/lumbar CSF ratio; the protein concentration measured in ventricular CSF is divided by the measured protein concentration in lumbar CSF. R/C = rostral/caudal ratio; serially collected lumbar CSF, the protein concentration ratio of the last fraction (most rostral) divided by the first fraction of collected lumbar CSF is shown. Brain-derived proteins: Tau, Microtubule-associated protein tau; S100B, Protein S100-B; NSE, Gamma-enolase; uniprot IDs: P05060, Secretogranin-1; Q06481, Amyloid-like protein 2. Blood-derived protein: Albumin, Serum albumin*.

* *Values derived from Brandner et al. (2014), intra-individual sample pairs were used*.

*I *Samples were obtained from patients suffering from NPH, lumbar CSF was collected 2 days before ventriculostomy and ventricle CSF during surgery*.

*II *Ventricle and lumbar CSF samples of patients suffering from posttraumatic hydrocephalus (PTH) were collected simultaneously at least 8 days after the last surgical or traumatic procedure*.

** *Values derived from Reiber (2001), lumbar CSF samples were taken for routine analysis, ventricle CSF samples were extracted from a drainage from not precisely specified collectives*.

*** *Values derived from Mollenhauer et al. (2012), serially collected lumbar CSF fractions (30–35th ml/1–5th ml) of five NPH patients*.

**** *Values derived from Aasebø et al. (2014), serially collected lumbar CSF fractions (44–45th mL/1–2nd ml) from a patient suffering from progressive supranuclear palsy (PSP); for reasons of representation the sample ratios were reversed in contrast to the original study. The samples were investigated by mass spectrometry, and quantified by iTRAQ, typical brain-derived proteins showed a ratio ~1, blood-derived proteins <1, no proteins with a considerably reversed gradient were detected indicating that the concentration of brain-derived proteins remain constant in the spinal CSF*.

It is noteworthy that different barrier permeabilities and/or bulk flow velocities of CSF along its course do not influence the outcome of the connected steady state model, since ͽ refers to the resulting mean barrier permeability in one individual and t to the resulting total flow time (see Equation 10).

Another possibility is that ventricle CSF is not only “young” CSF produced by the CP but also interstitial fluid from the highly perfused brain parenchyma, which already contains blood-derived proteins, see for instance Section 4.1 in Hladky and Barrand (2014) for a more detailed discussion.

The third possibility, an up-mixing of CSF from SAS back into the ventricles, is rather unlikely in normal physiological conditions and the diffusional fluxes are too low. However, most ventricle CSF samples described in the literature (summarized in Table 3) are derived from patients suffering from normal pressure hydrocephalus (NPH). In these patients retrograde flow has been observed (Penn et al., 2011). Possibly, the rostrocaudal gradient of blood-derived proteins tends to be less steep in NPH patients than in healthy individuals but this was not conclusive from the existing experimental data (see Table 3). Clearly, the discussed aspects need further experimental elucidation.

## The rostrocaudal gradient

The rostrocaudal gradient of blood-derived proteins suggests that the CSF bulk flow is the dominant process in explaining protein abundancies in CSF. However, as the bidirectional pulsatile flow velocity of CSF is considerably greater than bulk flow of CSF, and the geometry of the spinal SAS does not precisely resemble a straight cylinder and contains obstacles to flow such as at nerve roots (Pahlavian et al., 2014), it is plausible to assume that the pulsatile flow has an impact on the rostrocaudal gradient although the flow is predominantly laminar. Further, it was shown previously that pulsatile flow characteristics of CSF in the spinal canal influence the dispersion of intrathecally delivered drugs (Hettiarachchi et al., 2011) and change with activity (Edsbagge et al., 2004). In a study comparing the rostrocaudal gradient of total protein concentrations between young adults and elderly people, a roughly twofold higher CSF production rate and a ~25% (non-significant) higher protein rostrocaudal gradient were found in the younger group. In contrast, the total protein concentration in the older group was significantly higher than in the younger group (~59 mg^*^dl^−1^ compared to ~46.8 mg^*^dl^−1^) (May et al., 1990). A possible explanation for this discrepancy—a higher total protein concentration in the older group but a higher rostrocaudal gradient in the younger group—is the pulsatile flow of CSF. The lower turnover in the older group increased the mixing rate concomitantly with a decreased rostrocaudal gradient. Possibly, the compliant reaction to cranial blood flow was also diminished in the older cohort and therefore the peak flow amplitude of the pulsating CSF was higher, enhancing the mixing of spinal CSF as well. In conclusion, the total protein concentration is influenced by CSF bulk flow but the spinal rostrocaudal gradient of blood-derived proteins is also influenced by pulsatile flow characteristics of CSF.

It would be interesting to see experiments investigating the rostrocaudal gradient of blood-derived proteins in diseases such as Chiari malformation in which the flow path is hindered and CSF pulsatility is increased (Haughton et al., 2003; Wagshul et al., 2011). It was further shown that in patients suffering from Chiari malformation, turbulent flow pattern occurs (Helgeland et al., 2014). These altered CSF flow characteristics should be detectable by a decreased or breakdown of the rostrocaudal gradient of blood-derived proteins. However, spatial distances between altered flow patterns and lumbar puncture must be taken into account.

## Brain-derived proteins in CSF

The barrier between brain and CSF is considered to be leaky (Cserr, 1971), therefore uptake of brain proteins from the brain parenchyma into the CSF system might be dependent on diffusion and/or bulk flow; reviewed in Cserr (1971). This is also supported by various proteomic studies performed with lumbar CSF, showing that the lumbar proteome contains a CNS-derived fraction, for instance (Guldbrandsen et al., 2014; Zhang et al., 2015).

Further, the few clinically established brain-derived biomarkers measured in lumbar CSF such as S100B, amyloid-beta 42 or tau (Michetti et al., 1980; Blennow et al., 2001) indicate that brain-derived cranial protein uptake into CSF is reflected in lumbar CSF. Tracer studies suggest that the extent to which ISF is drained into the Cisterna magna (upstream of lumbar CSF) is different for different brain regions (Cserr et al., 1981; Szentistványi et al., 1984; Yamada et al., 1991). However, the intriguing question as to how well suited different brain areas are for readouts in lumbar CSF is beyond the scope of this work.

Here we simply state that the uptake of brain-derived substances in CSF is dependent on the exchange rate multiplied by the exchange time and therefore assume a dependency on CSF flow characteristics. Surprisingly, Reiber showed that brain-derived proteins do not correlate to Q_Alb_ in lumbar CSF which is used in CSF proteomics as an indicator of CSF flow characteristics. He suggested that the spatial limited uptake of brain-derived proteins into the CSF system is compensated by diffusional loss, as indicated by the distinctly higher ventricular than lumbar concentration of brain proteins (Reiber, 2001).

However, if increased uptake of brain-derived information is counterbalanced by diffusional loss, lumbar CSF-based diagnosis must be highly inaccurate. The extent of diffusion-out for brain proteins can be estimated by observing the diffusion rates of blood-derived proteins. The physiological normal Q_Alb_ in lumbar CSF is around 2–8 [10^−3^] meaning that only 0.2–0.8% of total albumin diffuses from serum into CSF. In conclusion, diffusional loss of brain proteins in the size range of albumin can be neglected but might be an aspect for small proteins or in CSF samples with very high Q_Alb_ values. Intriguingly the brain proteins Reiber presented, S100-B, Tau, NSE (gamma enolase), are all intracellular proteins (Reiber, 2001). Therefore, using Q_Alb_ as a reference protein might be biased since Q_Alb_ represents a different source (blood) and the uptake is dependent on cranial and spinal flow characteristics, whereas the uptake of brain-derived proteins predominantly takes place in the cranial space. Further, the reasons why these intracellular proteins are abundant in extracellular fluids is most likely independent of CSF flow characteristics.

These aspects and the fact that the Q_Alb_ represents not only CSF flow characteristics but also blood to CSF barrier characteristics might explain the lacking correlation of Q_Alb_ to the lumbar CSF concentrations of the investigated intracellular brain-derived proteins. Nevertheless, based on the expected common dependency to CSF flow characteristics we expect a certain degree of correlation between Q_Alb_ and brain-derived proteins if the sample size is sufficiently large.

Another important aspect is that under the assumption of bulk-flow (ventricular CSF mostly upstream and lumbar CSF mostly downstream) and diffusional loss being a very minor factor, the stoichiometry of the lateral ventricular to lumbar CSF protein concentration ratios for the investigated brain-derived proteins presented by Reiber (Table 3, 2nd column) and the general conclusion that brain-derived proteins are higher in ventricular than lumbar CSF is hard to explain (Reiber, 2001).

Net fluid introduction into the CSF system past the lateral ventricle CSF and therefore dilution to such an extent that brain-derived proteins are several times higher in lateral ventricle CSF than in lumbar CSF is unlikely considering the gross anatomic relations of the ventricular system; lateral ventricles make up ~80–90% of the ventricular system (Akdogan et al., 2010), and the corresponding ratio of blood-derived proteins (Table 3).

With an intelligent experimental design Brandner et al. showed that the high levels of brain-derived proteins in ventricular CSF compared to lumbar CSF is an effect of surgical interference (Brandner et al., 2013, 2014) releasing intracellular proteins in ventricular CSF and that the ventricular to lumbar CSF ratio is around 1 when the ventricular CSF withdrawal is performed sufficiently long after the traumatic surgery procedure (Table 3, 1st column).

These findings concur well with the assumption of bulk flow and the relative stable spinal protein concentration of brain-derived proteins along the rostrocaudal axis (Table 3).

Again, based on the limited sample and study numbers, the discussed aspects need further experimental elucidation.

In our view, the assumed dependency of brain-derived proteins on CSF flow characteristics introducing non-pathological individual variances questions the dominating biomarker discovery strategy based on the comparison of absolute concentration differences. We claim that the diagnostic accuracy can be improved by evaluating relative concentration values with suitable reference proteins similar to the approach used for blood-derived proteins. This aspect is presumed to be of special importance for early diagnosis purposes when subtle concentration changes of diseased to non-diseased individuals can be expected.

## Conclusions

The connected steady state model offers a solution for barrier-restricted diffusion between a continuous regenerating fluid system such as blood, and a river-like start/end system such as CSF. The model might have the potential to be a general solution concept for diffusional exchange of blood to other body fluids such as blood to the urine or lymph. The model fits perfectly to the available experimental data and is able to explain all the experimental findings. For our considerations we used experimental data already interpreted with the hyperbolic function (Reiber, 1994a). This might be an issue when interpreting the contradictory results at low Q_Alb_ values (see Section Discussion about the diffusion barriers and Appendix Figure 5A). So far we have described the diffusion through the barrier with a hydrodynamic radius-dependent factor and ignored other aspects such as biochemical properties of the proteins. This might be sufficient for soluble blood proteins but not for lipophilic substances. The availability of more experimental data and a more precise physiological understanding of how diffusion occurs will help to clarify these issues.

The source-related rostrocaudal gradient of proteins in CSF and the possibility to link the rostrocaudal gradient of blood-derived proteins to pulsatile flow characteristics are highly intriguing and so far underestimated aspects in CSF physiology but worthy of being investigated in more detail in the near future.

In spite of the enormous diagnostic potential of brain-derived proteins in CSF, their dependency on CSF flow characteristics is not sufficiently clarified. We assume that a better understanding and a better implementation of CSF dynamics in biomarker discovery approaches is the way toward the development of optimized analyzing strategies of the CSF-Proteome consequently leading to the identification of more sensitive and reliable brain-derived biomarkers.

## Author contributions

FM designed the study, was involved in all tasks and wrote the paper. DM contributed to the physical aspects of the study and to the discussion of the mathematical aspects of the study (Appendix A1). FS carried out the mathematical implementation of the model (Appendix A1) and contributed to the discussion of the physical aspects of the study.

### Conflict of interest statement

The authors declare that the research was conducted in the absence of any commercial or financial relationships that could be construed as a potential conflict of interest. The reviewer KB declared a shared affiliation, though no other collaboration, with several of the authors FHM, FS to the handling Editor, who ensured that the process nevertheless met the standards of a fair and objective review.
