# Supplementary material for: The Connected Steady State Model and the Interdependence of the CSF Proteome and CSF Flow Characteristics
Source: Front Neurosci. 2017 May 19;11:241. doi: 10.3389/fnins.2017.00241 (PMC5437178; doi:10.3389/fnins.2017.00241)
Supplement: Supplementary file 1 [file Appendix.pdf]

Appendix

A1) Mathematic formulation of the connected steady state system

For our derivation we start with event-based argumentation. The general expression is:

$$F_n = \beta\,(C_B - C_n) \qquad (1a)$$
$$C_{n+1} = C_n + k^{'} F_n \qquad (1b)$$

where $\beta := \mathfrak{I} * L^{-1}; k^{'} := \frac{1}{v};\ C_B := C_{blood}$ and $\mathrm{n} \in \mathbb{N}$. Substituting (1a) into (1b) yields:

$$C_{n+1} = C_n + \Delta\,(C_B - C_n) = C_n\,(1 - \Delta\,) + \Delta\,C_B.$$

where $\Delta := k^{'} * \beta$. Thus $(C_n)_{n \in \mathbb{N}}$ is an arithmetico-geometric sequence with the recursive relation:

$$C_{n+1} = aC_n + b$$

where $a := 1 - \Delta;\ b := \Delta *\ C_{B.}$ Observe that $0 < a < 1$ and $C_0 = 0$ (see Section Formulation of the Connected Steady State Model) and put $\mathrm{r} := \frac{b}{1-a} = C_B$. It is well known that $(C_n)_{n \in \mathbb{N}}$ admits the explicit formula:

$$C_n = a^n(C_0 - r) + r = -ra^n + r.$$

Now let $k_N' := \frac{k'}{N}$ for fixed $\mathrm{N} \in \mathbb{N}$ and let $(C_{N,n})_{n \in \mathbb{N}}$, $(F_{N,n})_{n \in \mathbb{N}}$ be the corresponding sequences for the concentration and diffusional flux at time $\frac{n}{N}$.

As seen above we have

$$C_{N,n+1} = C_{N,n} + k_N^{'} F_{N,n}$$

such that

$$C_{N,n+1} = a_N C_{N,n} + b_N$$

where $a_N := 1 - \frac{\Delta}{N}$; $b_N := \frac{\Delta}{N} * C_B$, thus

$$C_{N,n} = (a_N)^n(C_0 - r_N) + r_N = -r_N(a_N)^n + r_N$$

where $r_N := \frac{b_N}{1-a_N} = C_B$.

Therefore, a given time $t = ns = \frac{tN}{N}$ (n being a real number) corresponds to $n * N$ events of length $\frac{1}{N}s$ so that we get by approximation for the exact concentration C(t):

$$C(t) = \lim_{N\to\infty} C_{N,n*N} = \lim_{N\to\infty} -C_B\left(1-\frac{k^{'}\beta}{N}\right)^{n*N} + C_B$$

$$= \lim_{N\to\infty} -C_B\left[\left(1-\frac{k^{'}\beta}{N}\right)^{N}\right]^{n} + C_B$$

$$= -C_B e^{-k^{'}\beta n} + C_B$$

$$= -C_B e^{-k\beta t} + C_B$$

with $k := \frac{k^{'}}{1s}$. This also implies the relation for the diffusional flux F(t) at time t

$$F(t) = \beta\big(C_B - C(t)\big) = \beta C_B\, e^{-k\beta t}$$

Seen on relative levels yields:

$$\frac{C_{CSF}(t)}{C_{blood}} = \frac{-C_B e^{-k\beta t} + C_B}{C_B} = -e^{-\frac{\ni}{L}kt} + 1 \qquad \text{A1}$$

And analogously for the molecular flux:

$$\frac{F_{CSF}(t)}{F_{initial}} = \frac{\beta C_B\, e^{-k\beta t}}{\beta\, C_B} = e^{-\frac{\ni}{L}kt} \qquad \text{A2}$$

A2) The conformity of the molecular flux theory and the connected steady state model to the hyperbolic function and their valid range

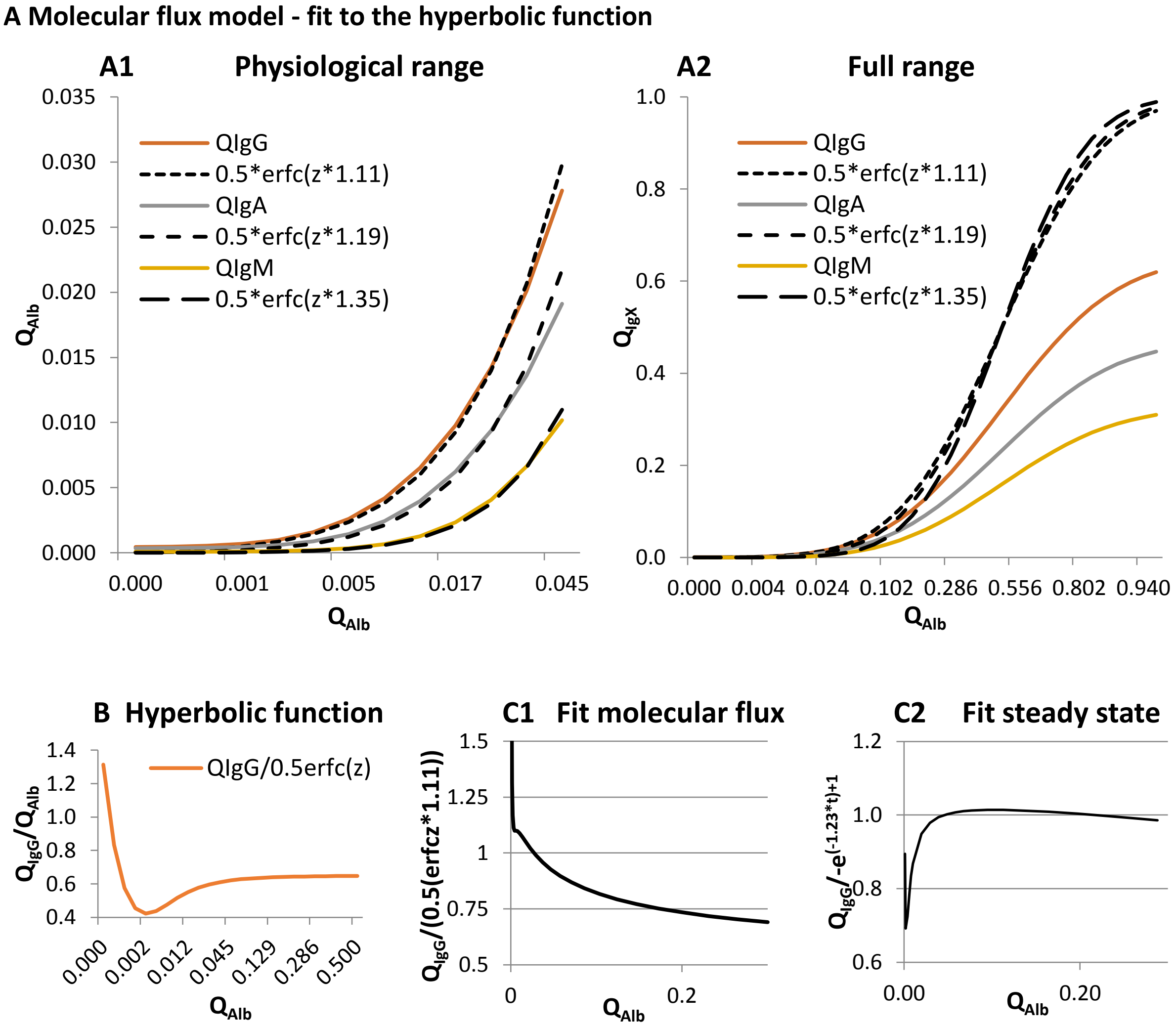

Fig. 5. Curve discussion. A: The function 0.5 erfc(z), $z = \frac{x}{2\sqrt{D_{Alb}t}}$, derived from the molecular flux model (Equation 3, Section The Molecular Flux Theory) fitted to the hyperbolic functions for IgG/A/M (Equation 1, Table 1). Q_{Alb} is represented by 0.5 erfc(z). The fitting procedure, see Equations A3/4, is the same as described for the connected steady state system (Section Validation of the Connected-Steady State Model, Equation 12/13).

A1: Like the connected steady state model (Figure 3B), the molecular flux model is also adjustable to the hyperbolic function in a certain physiological range.

A2: The fit shown over the whole range $0 \leq Q_{Alb} \leq 1$ shows several aspects: 1. Outside the overlapping range, the 0.5 erfc(z) function deviates considerably from the hyperbolic function. 2. At $Q_{Alb}<0.5$; $Q_{IgM}<Q_{IgA}<Q_{IgG}$ but at $Q_{Alb}>0.5$; $Q_{IgM}>Q_{IgA}>Q_{IgG}$. This does not meet the required boundary conditions (see Section The Molecular Flux Theory and Section The Connected Steady State Model)

B: The empirically derived hyperbolic function is only valid in the range in which the function is fitted to the experimental data**.** 0.5 erfc(z) is used for Q_{Alb} values (every other function increasing from 0 to 1 could be used as well). At high Q_{Alb} values (~ > 0.05) the function tends to a/b and not to 1; this can also be observed in **Figure5 A2** (also see Equation 1,

Table 1). However, theoretically at Q_{Alb}→1 Q_{IgX} must also tend to 1 and therefore $Q_{IgG}/Q_{Alb}=1$. At Q_{Alb}→ 0 the hyperbolic function (Equation 1) tends to a-c, resulting in $Q_{IgG} > Q_{Alb}$ which is impossible since Q_{Alb} is the faster diffusing molecule.
C1/2: Shows the accordance of the empirical hyperbolic function to the two theoretically derived functions.
C1: Shows that the function 0.5 erfc(z), representing the molecular flux theory, cannot be precisely fitted to the hyperbolic function.
C2: In the physiological range the connected steady state system fits precisely to the empirical function and therefore to the experimental data.

The approach to fit the equation derived from the ´molecular flux theory´ (Equation 3 in Section The Molecular Flux Theory) to the hyperbolic function (Equation 1) is the same as described for the connected steady state model (Section Validation of the Connected-Steady State Model, Equation 12/13). Equation A3 represents the theoretical function (Equation 3) and Equation A4 represents the empirical hyperbolic function (Equation 1).

$$\overline{Q_{IgX}} = \frac{1}{2} erfc\left(\frac{x}{2\sqrt{D_{Alb}t}}\sqrt{\frac{D_{Alb}}{D_{IgX}}}\right) = \frac{1}{2} erfc\left(z * \sqrt{\frac{D_{Alb}}{D_{IgX}}}\right) \qquad \text{A3}$$

$$\overline{Q_{IgX}} = \frac{a}{b}\sqrt{\frac{1}{2} erfc(z) + b^2} - c \qquad \text{A4}$$

with $z=\frac{x}{2\sqrt{D_{Alb}t}}$
